# Thermal Curing-Enhanced Circularly Polarized Phosphorescence

**DOI:** 10.3390/molecules31111967

**Published:** 2026-06-05

**Authors:** Shouchang Jiao, Rui Du, Jingcheng Wang, Hanlin Ou

**Affiliations:** Shandong Key Laboratory of Renewable Membrane Materials, College of Materials Science and Engineering, Qingdao University, Qingdao 266071, China; 15689019126@163.com (S.J.); dr99512021@163.com (R.D.); 15957538601@163.com (J.W.)

**Keywords:** room-temperature phosphorescence, circularly polarized phosphorescence, multicolor afterglow, anti-counterfeiting

## Abstract

Developing circularly polarized phosphorescence (CPP) materials integrating long-afterglow room-temperature phosphorescence (RTP) and chiral optical properties is highly attractive but challenging. Herein, we report a facile and efficient strategy to achieve enhanced CPP by doping chiral naphthyl phosphoric acid derivatives (BNP-CZ, BNP-DPA, BNP-TPA) into a thermally cured Bisphenol A Epoxy Resin (DGEBA) matrix crosslinked with 1,8-diaminooctane (DAO). The rigid crosslinked network effectively suppresses nonradiative transitions and stabilizes triplet excitons, affording a long phosphorescence lifetime of up to 973 ms and a high photoluminescence quantum yield of 26.55%. Significantly, the BNP-CZ@DAO exhibits remarkably boosted CPP signals and realizes the switch from circularly polarized fluorescence (CPF) in solution to CPP in the thermally cured resin film. Benefiting from the long afterglow and chiral optical properties, these polymers are successfully applied in multi-dimensional anticounterfeiting with high security. This work provides a universal and scalable approach for developing high-performance CPP materials.

## 1. Introduction

Organic room-temperature phosphorescence [1,2,3,4,5,6,7] (RTP) materials exhibit promising application potential in diverse fields including display technologies, information encryption, anticounterfeiting, organic light-emitting diodes, and bioimaging [8,9], owing to their remarkable advantages such as large Stokes shifts, long emission lifetimes, and excellent biocompatibility [10,11,12,13,14,15,16,17,18]. However, the inherently spin-forbidden nature of transitions from the excited singlet state to the triplet state generally results in low RTP efficiency [19,20,21]. In addition, long-lived triplet excitons are easily quenched by environmental factors such as water and oxygen, which pose a severe challenge to achieving persistent and intense phosphorescence emission [22,23,24,25]. To overcome these limitations, numerous strategies including heavy-atom strategy, crystallization, host-guest doping and supramolecular strategy [26] have been developed to enhance intersystem crossing (ISC) efficiency and alleviate triplet exciton quenching in organic RTP materials [27,28,29,30,31,32,33,34,35]. Among these approaches, the polymer doping strategy, which relies on the rigid microenvironment generated by van der Waals forces and hydrogen-bonding interactions within the polymer matrix to suppress nonradiative triplet energy dissipation, has attracted considerable research attention due to its merits of facile material preparation, favorable processability, and strong universality [36,37,38,39,40,41]. To date, various conventional polymers, including polymethyl methacrylate, polyvinyl alcohol, and sodium alginate, have been widely employed as polymer matrices for the construction of RTP materials [42,43]. However, most polymers impose a relatively weak confinement effect on luminescent molecules; novel polymer design strategies are required to strengthen such confinement and improve RTP performance.

Furthermore, circularly polarized luminescence [44] (CPL) materials featuring RTP characteristics, as a novel category of optical functional materials, have been extensively applied in information coding, anticounterfeiting, optoelectronics and other fields on account of their distinctive optical signals [45,46,47,48,49,50,51]. Circularly polarized phosphorescence (CPP) imaging based on such materials is endowed with higher sensitivity and lower background interference in comparison with conventional fluorescence imaging, thereby enabling clearer and more accurate imaging outputs to be obtained [52]. Meanwhile, these materials can be further utilized in the fabrication of products with specialized coding and anticounterfeiting functions [53]. Although considerable progress has been made by researchers in the development of CPP materials [54], relatively short emission lifetimes are still observed for conventional polymer-based long-afterglow CPP systems [55]. Consequently, the exploitation of facile strategies that integrate high efficiency, universality, and favorable capability for boosting CPL signals has emerged as an urgent requirement in current research.

**Figure 1:**
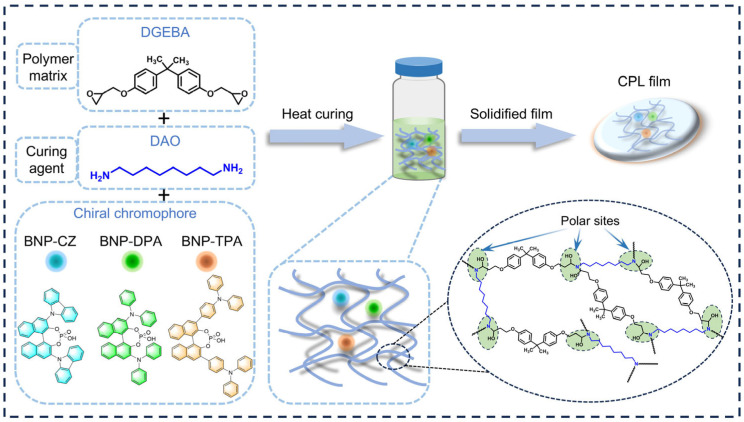
Schematic design strategy for thermal curing epoxy resin enhanced CPP based on doped chiral chromophores.

In recent years, the thermal curing strategy has emerged as an innovative approach to prolong the afterglow lifetime of RTP materials. However, the application of this strategy in constructing long-lived CPP materials has not yet been reported. Bisphenol A Epoxy Resin (DGEBA) possesses excellent film-forming ability, stability, and processability [56,57,58]. Its unique crosslinked network structure and remarkable mechanical properties show great potential to provide new strategies for simultaneously boosting RTP and CPP performances. Inspired by these advantages, we herein propose a facile and efficient thermal curing strategy to construct high-performance CPP materials, using chiral long-afterglow phosphorescent materials as emission centers, and thermosetting resins formed by the crosslinking of DGEBA with 1,8-diaminooctane (DAO) as the polymer matrix (Figure 1). Chiral naphthyl phosphoric acid derivatives (BNP-CZ, BNP-DPA, and BNP-TPA) were confined within the network at a chromophore-to-polymer mass ratio of 1:500 (Table S1), affording three chiral polymeric films (BNP-CZ@DAO, BNP-DPA@DAO, and BNP-TPA@DAO) with ultralong organic phosphorescence (UOP) properties. Abundant polar sites in the matrix can provide sufficient intermolecular interactions, which facilitate the ISC process and the generation of triplet excitons [6,59,60,61,62]. Meanwhile, the chromophore motions are effectively restricted in the rigid matrix, suppressing nonradiative transition [60,63,64,65]. Notably, the rigid thermally cured epoxy network enables the transformation from CPF dominance before thermal curing to CPP dominance after thermal curing, distinguishing this work from previous polymer-based CPL systems. Meanwhile, the obtained polymer exhibits comparable CPP properties to those reported in previous work (Table S2) [66,67,68,69]. This work provides a new and reliable pathway for the construction of high-performance CPP materials.

## 2. Results and Discussion

### 2.1. Characterization of the Photophysical Properties of Chiral Phosphoric Acid Molecules

**Figure 2:**
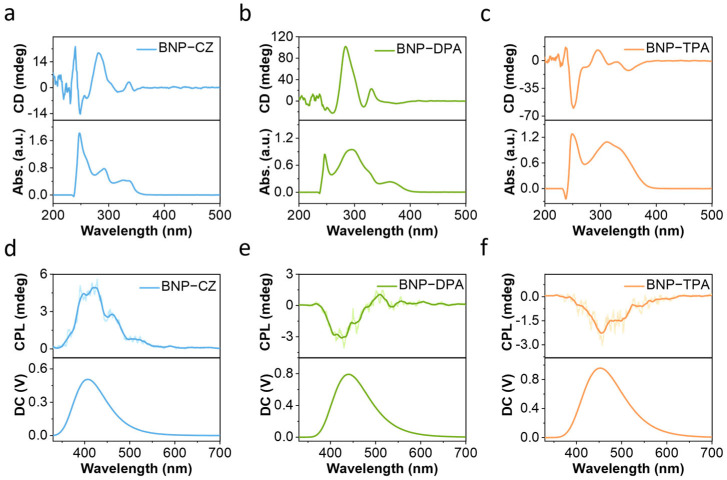
Photophysical properties of chiral naphthylphosphoric acid derivatives in DMSO solution. (**a**) CD spectra and (**d**) CPL spectra of BNP-CZ in DMSO solution. (**b**) CD spectra and (**e**) CPL spectra of BNP-DPA in DMSO solution. (**c**) CD spectra and (**f**) CPL spectra of BNP-TPA in DMSO solution (λ_ex_ = 312 nm).

Firstly, the photophysical properties of three chiral naphthyl chromophores (BNP-CZ, BNP-DPA, and BNP-TPA) were systematically investigated. The UV-Vis absorption and prompt photoluminescence (PL) spectra of these chromophores were characterized in dilute DMSO solutions. All chromophores exhibited distinct absorption features in the range of 250–375 nm (Figure S1a–c), suggesting their broad-spectrum absorption ability. Notably, upon excitation at 254, 312, and 365 nm, these compounds displayed characteristic fluorescence emission peaks at 425–454 nm (Figure S1d–f), which are consistent with the blue-violet fluorescence emission. Subsequently, circular dichroism (CD) and CPL spectra were recorded to evaluate the chiroptical properties of BNP-CZ, BNP-DPA, and BNP-TPA in DMSO solution. As depicted in Figure 2, all three chromophores exhibited intense CD (Figure 2a–c) and CPL signals at room temperature. Specifically, the CPL spectrum of BNP-CZ showed a positive signal (Figure 2d), whereas BNP-DPA and BNP-TPA exhibited negative CPL signals (Figure 2e,f). The solution-state luminescence dissymmetry factors (|glum|) of BNP-CZ, BNP-DPA, and BNP-TPA are determined to be 0.56 × 10^−3^, 0.47 × 10^−3^, and 1.29 × 10^−3^, respectively, showing weak chiroptical responses in dilute solution (Table S3). The CPL signal corresponds well to the fluorescence emission peaks of each chromophore in solution. In the range of 250–400 nm, the CD profiles of these chromophores matched well with their absorption bands in dilute DMSO solution. In the solid powder state, BNP-CZ exhibited an intense blue structureless emission band at 425 nm, while BNP-DPA and BNP-TPA showed similar strong blue emission bands around 454 nm, with fluorescence lifetimes of 14, 7.5, and 10.8 ns, respectively (Figure S2), demonstrating typical fluorescence characteristics.

### 2.2. Photophysical Properties of Doped Films

The photophysical properties of the undoped resin were characterized. As shown in Figure S3, the prompt PL spectrum of the resin cured with DAO and 1-nonanamine (1-NA) exhibited an obvious redshift. Specifically, as the excitation wavelength increased, the emission peak shifted from 310 nm to 420 nm, showing a gradual redshift trend. Meanwhile, this result is consistent with the blue fluorescence images shown in the figure, confirming the typical fluorescence emission behavior of the resin. Subsequently, the influence of curing agent 1-NA on the crosslinking degree of the polymer network was systematically investigated. To this end, three polymers were prepared: BNP-CZ@1-NA, BNP-DPA@1-NA, and BNP-TPA@1-NA, where the mass ratio of the chiral molecules (BNP-CZ, BNP-DPA, BNP-TPA) to DGEBA was fixed at 1:500. As shown in Figures S4 and S5, the long-wavelength emission peaks in the prompt PL spectra of the polymer films in the DAO and 1-NA systems exhibited no obvious redshift with varying excitation wavelengths, indicating that the crosslinking degree of the polymer network had little influence on the characteristics of the prompt PL spectra. Furthermore, the delayed emission spectra of the polymer films in the DAO system were analyzed. As shown in Figure S6, the emission peak positions of BNP-CZ@DAO, BNP-DPA@DAO, and BNP-TPA@DAO showed no obvious changes under different excitation wavelengths. Specifically, the emission peaks of these polymers were located at 545 nm, 560 nm, and 560 nm, respectively. Notably, the doped films in the DAO system exhibited the highest emission intensities under 312 nm excitation. Among them, the phosphorescence emission intensities of the BNP-TPA@DAO film under 312 nm and 365 nm excitation showed no significant difference, whereas the intensities of all polymer films decreased sharply under 254 nm excitation.

**Figure 3:**
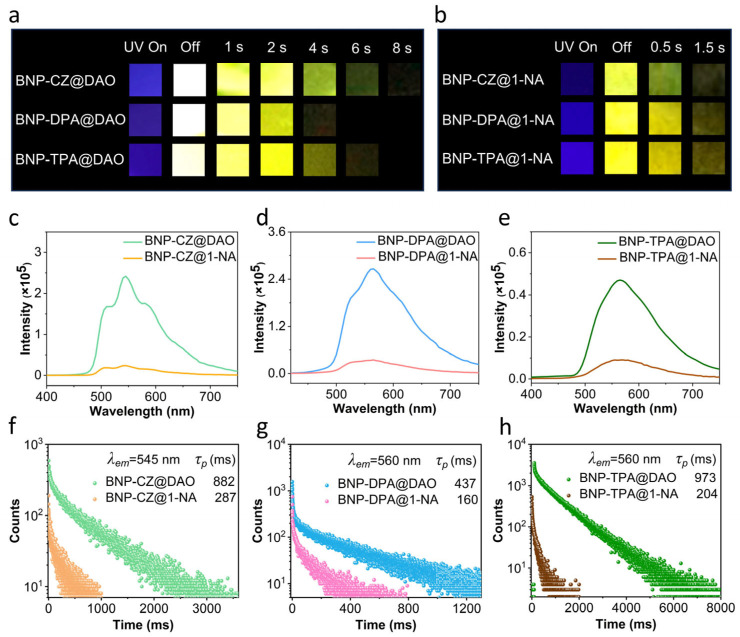
The photographs are taken under and after removing the 312 nm UV irradiation of (**a**) DAO-doped system and (**b**) 1-NA-doped system polymer films. (**c**–**e**) Delayed PL spectra (t_d_ = 1 ms, λ_ex_ = 312 nm) of BNP-CZ@DAO, BNP-DPA@DAO, and BNP-TPA@DAO, respectively. (**f**) Time-resolved emission spectra (λ_ex_ = 312 nm) of BNP-CZ@DAO and BNP-CZ@1-NA. (**g**) Time-resolved emission spectra (λ_ex_ = 312 nm) of BNP-DPA@DAO and BNP-DPA@1-NA. (**h**) Time-resolved emission spectra (λ_ex_ = 312 nm) of BNP-TPA@DAO and BNP-TPA@1-NA.

By introducing curing agents with amino groups into the epoxy resin matrix, the oxirane rings at both ends of the epoxy resin underwent ring-opening reactions with the amino groups of the curing agents, and gradually crosslinked to form a compact polymer network under heating, indicating that uniform and dense amorphous films were formed after curing. As shown in Figure 3, under 312 nm UV irradiation, the obtained polymers BNP-CZ@DAO, BNP-DPA@DAO, and BNP-TPA@DAO exhibited bright blue emission in the solid-state film. After ceasing the irradiation, a visible yellow afterglow of up to 8 s could be observed under ambient conditions (Figure 3a), and the PL quantum yields of these three polymer films were determined to be 5.94%, 20.22%, and 26.55%, respectively (Table S4). The DAO-based and 1-NA-based polymer films showed identical prompt and delayed PL spectra, which could be ascribed to the same chiral chromophores in these polymers. When the curing agent was changed from DAO to 1-NA, with a time delay of 1 ms, the same yellow emission band could be clearly detected under 312 nm excitation, with the maximum emission peaks located at 545 nm and 560 nm, respectively. Meanwhile, the afterglow duration of the obtained polymer films decreased from 8 s to 1.5 s (Figure 3a,b). Among them, the longest excited-state lifetime of the BNP-TPA@DAO film was calculated to be 973 ms using the triple-exponential fitting formula: $R_{\left(t\right)}=B_{1}e^{\left(-t/\tau_{1}\right)}+$ $B_{2}e^{\left(-t/\tau_{2}\right)}+\;B_{3}e^{\left(-t/\tau_{3}\right)}$, showing remarkably long-lived RTP behavior (Table S5) [12]. It can be concluded that under the same conditions, the phosphorescence intensity (Figure 3c–e), afterglow duration, and phosphorescence lifetime (Figure 3f–h) of the polymer films in the 1-NA system were all lower than those in the DAO system. This result demonstrates that the increased number of terminal amino groups in the curing agent chain significantly enhances the rigidity of the polymer network and suppresses the exciton quenching process. Further, Differential Scanning Calorimetry (DSC) measurements were performed to characterize the thermal properties of the crosslinked networks. As shown in the DSC curves, the glass transition temperature (T_g_) of the DGEBA@DAO network is significantly higher than that of the DGEBA@1-NA system. Specifically, the T_g_ of DGEBA@DAO is determined to be 80 °C, while the DGEBA@1-NA network exhibits a much lower T_g_ (−10 °C). This clear difference directly confirms that the DAO-crosslinked network possesses a higher crosslink density and a more rigid microenvironment, which is consistent with our proposed mechanism. Furthermore, Dynamic Mechanical Analysis (DMA) measurements were also performed to validate this conclusion. The results show that the DGEBA@DAO system exhibits a higher storage modulus (E′) and a higher T_g_ (determined from the tan δ peak) compared to DGEBA@1-NA, providing complementary mechanical evidence for the enhanced rigidity (Figure S7). The molecular motions were effectively restricted via the additional multiple intermolecular interactions between the chiral chromophores and the rigid polymer network. Meanwhile, the quenching of triplet excitons was also significantly suppressed, which could be attributed to the rigid and compact environment provided by the cured and crosslinked polymer network. To confirm the curing reaction of DGEBA@DAO system, Fourier Transform Infrared (FTIR) spectra before and after thermal curing were recorded. After curing, a significant enhancement of the broad absorption band at ~3450 cm^−1^ was observed, which is attributed to the stretching vibration of newly formed hydroxyl (-OH) groups generated by the ring-opening reaction of epoxy groups. Meanwhile, the characteristic absorption peaks corresponding to epoxy groups, including the C-O-C asymmetric stretching vibration of the oxirane ring at ~915 cm^−1^, the C-O stretching vibration at ~1250 cm^−1^, and the ring deformation vibrations at ~750–850 cm^−1^, disappeared completely after curing. These results provide direct evidence for the consumption of epoxy groups and the formation of a crosslinked network, which is consistent with the proposed curing mechanism (Figure S8). Besides, the afterglow photographs of the polymer film under nitrogen and air atmospheres show negligible difference in afterglow duration, indicating that the dense structure formed after thermal curing effectively hinders oxygen penetration into the film.

Under excitation with 254 nm ultraviolet (UV) light, the polymer films BNP-CZ@DAO, BNP-DPA@DAO, and BNP-TPA@DAO all exhibited bright blue fluorescence emission. Notably, after the removal of the UV light source, a yellow afterglow phenomenon lasting approximately 4 s could be observed for these materials. Control experiments showed that under 254 nm excitation, the afterglow duration and afterglow lifetime of the films were all significantly lower than the corresponding values under 312 nm and 365 nm excitation (Figure S10). Further studies revealed that compared with the DAO-based polymers, the 1-NA-based polymer system exhibited an obvious downward trend in afterglow performance under 254 nm excitation. Specifically, the afterglow duration was shortened from 4 s to approximately 1.5 s (Figure S11a), and the maximum phosphorescence lifetime was also reduced from 378 ms to 116 ms (Figure S11c–e). However, interestingly, despite the decrease in afterglow performance, the positions of the main emission peaks of the three 1-NA-based polymers (BNP-CZ@1-NA, BNP-DPA@1-NA, and BNP-TPA@1-NA) remained stable, with their phosphorescence emission peaks located at 545 nm, 560 nm, and 560 nm, respectively (Figure S11b), without an obvious shift.

Furthermore, the present study revealed that when the excitation wavelength was switched to 365 nm, the polymer films still exhibited obvious long-lived afterglow luminescence. Among them, the BNP-TPA@DAO film showed the best performance, with a yellow afterglow duration of up to 8 s (Figure S12) and a maximum afterglow lifetime of 887 ms. In contrast, the afterglow durations of BNP-CZ@DAO and BNP-DPA@DAO films were slightly shorter, at approximately 6 s. Notably, these polymer films exhibited favorable RTP behavior under different UV excitations. Analysis of the structure-property relationship indicates that the broad-spectrum response of RTP is primarily attributed to the unique axial chiral structure of the chiral phosphate molecule. First, the conjugated structure of the chiral molecules effectively promoted the ISC process. Second, the rigid molecular skeleton and the rigid microenvironment provided by the polymer matrix suppressed nonradiative transitions. In addition, the broad UV absorption of chiral phosphoric acid molecules in the range of 250–375 nm allowed effective excitation at different wavelengths. The synergistic effect of these structural features enabled the materials to achieve efficient phosphorescence emission under various UV excitation conditions.

Compared with the DAO polymer system, the polymer films BNP-CZ@1-NA, BNP-DPA@1-NA, and BNP-TPA@1-NA in the 1-NA polymer system exhibited obvious yellow emission bands under a time delay of 1 ms and excitation at 365 nm (Figure S13), with the maximum emission peaks located at 545 nm, 560 nm, and 560 nm, respectively. This result was highly similar to the delayed emission spectra of the corresponding DAO polymer system, indicating that the emission bands of the 1-NA polymer series at the corresponding wavelengths could be attributed to phosphorescence emission. Among them, BNP-TPA@1-NA exhibited the longest afterglow lifetime of approximately 310 ms. Moreover, it could be clearly observed that under 254 nm and 365 nm excitations, the yellow emission intensity near the phosphorescence emission peaks of the 1-NA polymer system was significantly reduced, accompanied by decreased measured phosphorescence lifetimes (Figure S14). This indicated that the 1-NA curing agent exerted a significant influence on the rigidity of the polymer network, and the rigid environment was closely related to the RTP performance. A favorable rigid environment could effectively restrict molecular motion, thereby suppressing nonradiative transitions. Notably, in the polymer film system, the phosphorescence emission behavior mainly originated from the isolated chromophore molecules dispersed in the polymer matrix. The polymer matrix itself did not directly participate in the luminescence process, but exerted a spatial confinement effect on the doped chromophore molecules through its unique rigid network structure. Specifically, this confinement effect was manifested as follows: on the one hand, the crosslinked network formed by the long polymer chains could effectively inhibit the vibration and rotation of chromophore molecules, significantly reducing the nonradiative transition rate; on the other hand, the microenvironmental barrier provided by the polymer matrix could block the quenching of triplet excitons by quenchers such as oxygen and moisture.

### 2.3. Circularly Polarized Properties of DAO System Doped Films

**Figure 4:**
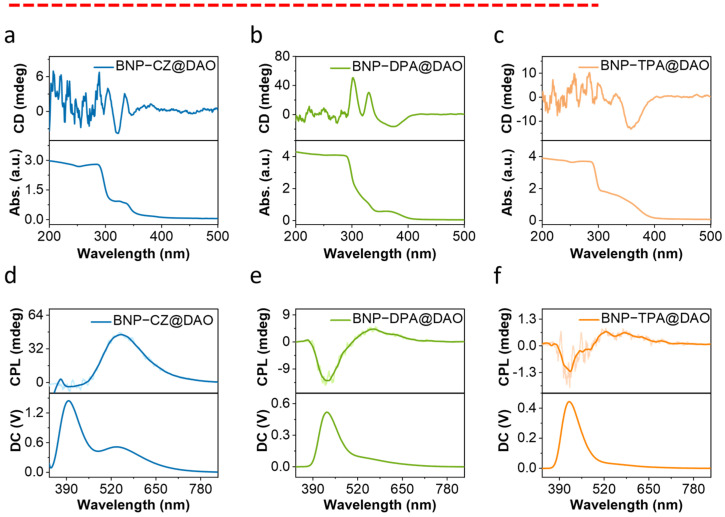
(**a**) CD spectra and (**d**) CPL spectra of BNP-CZ@DAO doped film. (**b**) CD spectra and (**e**) CPL spectra of BNP-DPA@DAO doped film. (**c**) CD spectra and (**f**) CPL spectra of BNP-TPA@DAO doped film (λ_ex_ = 312 nm).

After confirming the UOP properties of the polymer films, CD and CPL spectroscopy were employed to investigate the chiroptical properties of the chiral polymers BNP-CZ@DAO, BNP-DPA@DAO, and BNP-TPA@DAO. As depicted in Figure 4a–c, all three films in the DAO system exhibited intense CD and CPL signals at room temperature. In the range of 250–350 nm, the CD profiles and absorption spectra of the polymer films coincided well with those of the chiral chromophores in dilute DMSO solution, indicating that the chiral chromophores retained characteristic CD signals and maintained their chiral configuration within the polymer matrix. Impressively, the CPL spectrum of the BNP-CZ@DAO film displayed a distinct positive signal peak at 545 nm (Figure 4d), which matched the corresponding phosphorescence emission peak in the solid state. In contrast, the CPL signal maximum of BNP-CZ in dilute DMSO solution corresponded to its fluorescence emission peak. After thermal curing, the BNP-CZ@DAO film shows no obvious enhancement in CPF. Nevertheless, comparison of the samples before and after thermal curing reveals that the cured film presents 100.2 times enhanced CPP intensity and a larger |glum| (Figure S15, and Table S4). This observation demonstrated that the rigid polymer network triggered the conversion of the CPL signal of BNP-CZ from fluorescence to phosphorescence, achieving enhanced CPP intensity. Notably, there is no chirality transfer that takes place in the system, and the measured CPP signals are derived exclusively from the intrinsic chirality of the embedded chiral luminophores. The substantial increase in CPP intensity upon thermal curing is attributed to the enhanced rigidity of the cured matrix, which restricts molecular motions, suppresses nonradiative relaxation, and thereby boosts the inherent CPP performance of the chiral molecules without invoking any chirality transfer mechanism. Nevertheless, the CPL spectra of BNP-DPA@DAO and BNP-TPA@DAO films show a distinct negative signal peak at 440 nm, while only a weak response appears around 560 nm (Figure 4e,f and Table S4). This profile matches well with the maximum fluorescence emission of the polymer films. Considering that the prompt PL spectra of these samples exhibit negligible phosphorescence contributions, the observed dissymmetric luminescence is dominated by CPF, while the contribution from CPP is relatively weak.

### 2.4. Optical Applications in Anticounterfeiting

**Figure 5:**
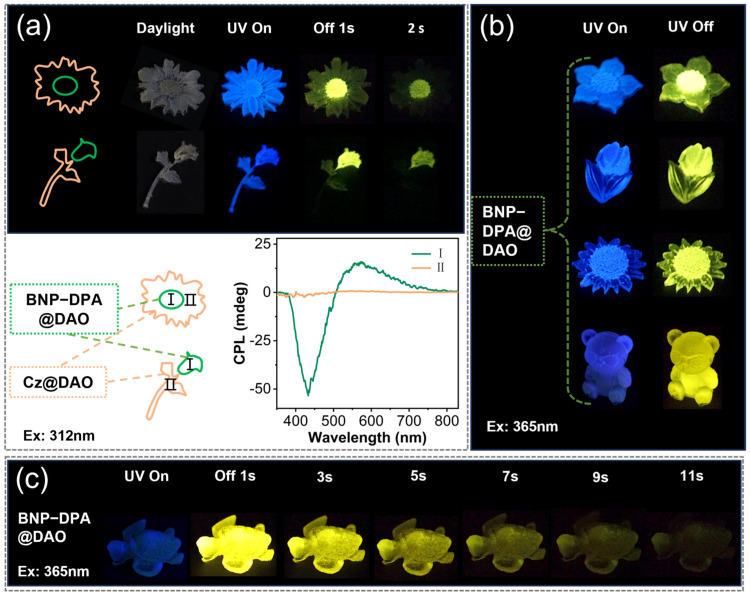
Multiple anticounterfeiting applications of heat-curable resin-based polymers. (**a**) Flower-shaped polymer anticounterfeiting pattern. Different CPL signals can be detected in different parts of the flower. (**b**,**c**) Blue fluorescence of polymers with different patterns when excited at 365 nm and yellow ultralong afterglow after turning off the excitation (Cz: 9H-carbazole-2,7-dicarboxylic acid).

The excellent luminescent properties of thermally cured resin films, including RTP, CPF, CPP, multicolor emission, and unique temporal response, endow them with great potential for advanced applications. Inspired by these distinctive optical features, the feasibility of an anticounterfeiting system was explored. Given the good flexibility, transparency, long-lived organic phosphorescence, and CPP characteristics of the DAO-series polymers, their potential applications in anticounterfeiting were further investigated. In this work, an advanced anticounterfeiting system was successfully constructed by ingeniously integrating the CPP properties with the long-lived luminescence of the chiral chromophores. As shown in Figure 5a, well-defined anticounterfeiting patterns were fabricated using polymers BNP-DPA@DAO (I) and Cz@DAO (II), respectively. Upon UV excitation, different regions of the flower pattern exhibited similar bright blue fluorescence emission. However, after removing the UV light source, the petal region doped with 9H-carbazole-2,7-dicarboxylic acid displayed prompt emission quenching, whereas the stamen region composed of BNP-DPA@DAO exhibited persistent bright yellow afterglow. This remarkable distinction enabled a dual fluorescence-phosphorescence anticounterfeiting function. Notably, upon CPL detection, obvious CPL signals could only be observed in region (I), while region (II) exhibited no chiroptical response, thereby realizing a triple fluorescence-phosphorescence-circularly polarized luminescence anticounterfeiting system. This innovative design overcomes the limitations of conventional anticounterfeiting technologies. Compared with previous systems, the anticounterfeiting strategy proposed in this work exhibits remarkable superiority. Traditional anticounterfeiting approaches based on ultralong organic phosphorescent materials usually rely on a single optical dimension, such as lifetime coding or emission color coding, where anticounterfeiting is realized only by modulating the phosphorescence lifetime and emission color of different chromophores. In contrast, the present strategy significantly improves the complexity and security of multi-level anticounterfeiting systems through the synergistic effect of multi-dimensional optical properties, opening new avenues for high-end anticounterfeiting and related advanced applications.

As shown in Figure 5b,c, various shapes of anticounterfeiting marks were designed and prepared through steps such as modeling with a high-temperature resistant silicone mold, injecting thermally cured resin polymers doped with chromophores, heating and curing, and demolding. When excited by a 365 nm UV lamp, the prepared anticounterfeiting marks exhibited bright blue fluorescence emission. After stopping the UV excitation, these marks could continuously emit a visible yellow afterglow to the naked eye at room temperature, with a duration of up to 11 s. This ultralong afterglow property enables the anticounterfeiting marks to maintain observable optical signals without excitation source stimulation, greatly improving their anticounterfeiting performance and application potential. As shown in Figure S16, BNP-DPA@DAO still retains intense persistent luminescence after one year of storage (BNP-DPA@DAO-1) under ambient conditions, revealing superior long-term stability that satisfies practical anticounterfeiting requirements. Besides, upon immersion in water, the BNP-DPA@DAO-W sample emits comparative afterglow under UV irradiation. This indicates that encapsulation within a rigid epoxy matrix effectively shields luminescent substances from quenching induced by water and oxygen, proving its outstanding moisture resistance. Moreover, the material maintains steady, persistent luminescence across varying temperatures, validating the operational reliability of anticounterfeiting patterns in real-world applications. As displayed in Figures S17 and S18, the prompt and delayed emission spectra of BNP-DPA@DAO-1 and BNP-DPA@DAO-W are highly consistent with those of BNP-DPA@DAO, accompanied by negligible decay in luminescence lifetime. Due to the processability, good optical properties, and large-area preparation performance of such epoxy compounds, a series of potential applications based on polymer UOPs has been discovered, and these applications are expected to be further expanded and optimized due to the excellent optical sensitivity and spatial resolution of CPP materials.

## 3. Conclusions

In this work, a chiral molecule doping strategy was proposed based on the rigid structural characteristics of thermally cured resins, which successfully realized significant enhancement of the CPP signals of chiral emitters. Specifically, chiral phosphoric acid chromophores with yellow afterglow were incorporated into a DGEBA system, and films were fabricated by thermal curing with suitable curing agents, realizing the regulation of CPP signals in thermally cured resin-based RTP materials. By utilizing hydrogen bonds and other polar interactions between chiral phosphoric acid molecules and the epoxy resin matrix, long-lifetime thermally cured resin-based RTP materials with pronounced CPP characteristics were successfully prepared. Benefiting from the large conjugation structure of the chiral phosphoric acid units and the synergistic effect of multiple intermolecular interactions, the resultant RTP materials exhibited a maximum phosphorescence lifetime of 973 ms and a PL quantum yield of up to 26.55%. Systematic investigation of curing agents with different amino functionalities (DAO and 1-NA) revealed that the molecular structure of the curing agent exerted a significant regulatory effect on the RTP properties of the doped films. The DAO-crosslinked network (with terminal amino groups at both ends) possessed higher crosslinking density, providing a more rigid microenvironment for chiral molecules. In contrast, 1-NA (with a single terminal amino group) showed a weaker ability to restrict molecular motion due to its lower crosslinking density. Meanwhile, the DAO system exhibited significantly enhanced RTP intensity and more prominent CPP signals. This endowed BNP-CZ@DAO with favorable afterglow lifetime and CPP characteristics simultaneously, further broadening the application scope of organic RTP materials. Benefiting from the excellent long-afterglow RTP performance and CPP features of the chiral species in the thermally cured resin matrix, the obtained materials were successfully applied in anticounterfeiting. The strategy for enhancing CPP signals based on a thermally cured resin platform proposed in this study provides a new avenue for the development of high-performance CPP materials.

## Data Availability

Data are contained within the article and Supplementary Materials.

## Supplementary Materials

The following supporting information can be downloaded at: https://www.mdpi.com/article/10.3390/molecules31111967/s1.
