# Supplementary material for: Thermal Curing-Enhanced Circularly Polarized Phosphorescence
Source: Molecules. 2026 Jun 5;31(11):1967. doi: 10.3390/molecules31111967 (PMC13257652; doi:10.3390/molecules31111967)
Supplement: Supplementary file 1 [file molecules-31-01967-s001.zip › molecules-4313795-supplementary.pdf]

Supporting Information

Thermal Curing-Enhanced Circularly Polarized Phosphorescence

Shouchang Jiao †, Rui Du †, Jingcheng Wang, and Hanlin Ou *

Shandong Key Laboratory of Renewable Membrane Materials, College of Materials Science and Engineering, Qingdao University, Qingdao 266071, China.

* Correspondence: hlou@qdu.edu.cn.

† These authors contributed equally to this work.

Experimental

Reagents and materials. Unless otherwise stated, all commercially available chemicals were used without further purification. Bisphenol A Epoxy Resin (DGEBA) was obtained from Sigma-Aldrich Co., Ltd. 1,8-diaminooctane (DAO, 98%) and 1-nonanamine (1-NA, 99%) were purchased from Sigma-Aldrich Co., Ltd. and Bidepharm Reagent Co., Ltd., respectively. Chiral naphthyl phosphoric acid derivatives (BNP-CZ, BNP-DPA, BNP-TPA) were provided by the research group of Professor Zhongwen Wang, Nankai University. All other solvents and reagents were of analytical grade and used without further purification.

Instrumentation. UV-vis absorption spectra were recorded on a Shimadzu UV-3600Plus spectrophotometer at room temperature. Steady-state photoluminescence (PL), delayed PL, phosphorescence emission, and phosphorescence lifetime measurements were performed on an Edinburgh FLS 1000 fluorescence spectrometer at ambient conditions. Circular dichroism (CD) spectra were collected on a Jasco J-1500 spectropolarimeter. Circularly polarized luminescence (CPL) spectra were measured using a Jasco CPL-300 fluorometer. X-ray diffraction (XRD) patterns were acquired on a Bruker D8 ADVANCE Da Vinci diffractometer with Cu Kα radiation. All luminescence photographs were taken using a Sony α7m4 digital camera under irradiation with a portable UV lamp (EA-160/FC, Spectronics Co., USA) at 254 nm, 312 nm, and 365 nm.

Preparation of Chiral Phosphoric Acid@DAO Doped Films. Taking BNP-CZ as a typical example, BNP-CZ (0.8 mg) and DGEBA (400 mg) were added into a 5 mL vial. The mixture was stirred at 100 °C until BNP-CZ was completely dissolved, and then cooled to 90 °C. Curing agent DAO (80 mg) was added, and the system was stirred at a constant temperature for another 3 min to obtain a viscous prepolymer. The mixture was cast onto a quartz substrate and dried on a hot stage at 95 °C for 3 h to afford the BNP-CZ@DAO film. Chromophores BNP-DPA and BNP-TPA were doped into the DGEBA resin at the same mass ratio, respectively.

Preparation of Chiral Phosphoric Acid@1-NA Doped Films. Taking BNP-CZ as a representative example, BNP-CZ (0.8 mg) and DGEBA (400 mg) were placed into a 5 mL vial. The mixture was stirred at 100 °C until BNP-CZ was fully dissolved, followed by cooling to 90 °C. Subsequently, curing agent 1-NA (200 mg) was added to the homogeneous mixture of BNP-CZ and DGEBA, and stirring was continued at constant temperature for 3 min to yield a viscous prepolymer. The as-obtained mixture was cast onto a quartz substrate and dried on a hot stage at 95 °C for 3 h to afford the BNP-CZ@1-NA film. BNP-DPA@1-NA and BNP-TPA@1-NA films were prepared via the same procedure.

Preparation of Anti-counterfeiting Models. The mixture of DGEBA containing 0.2 wt% chromophore and the corresponding curing agent was heated and stirred at 90 °C. Before gelation, the mixture was poured into high-temperature-resistant molds with various patterns, followed by thermal curing at 95 °C for 3 h. Finally, a series of afterglow models with diverse shapes were obtained.

Figures and Tables

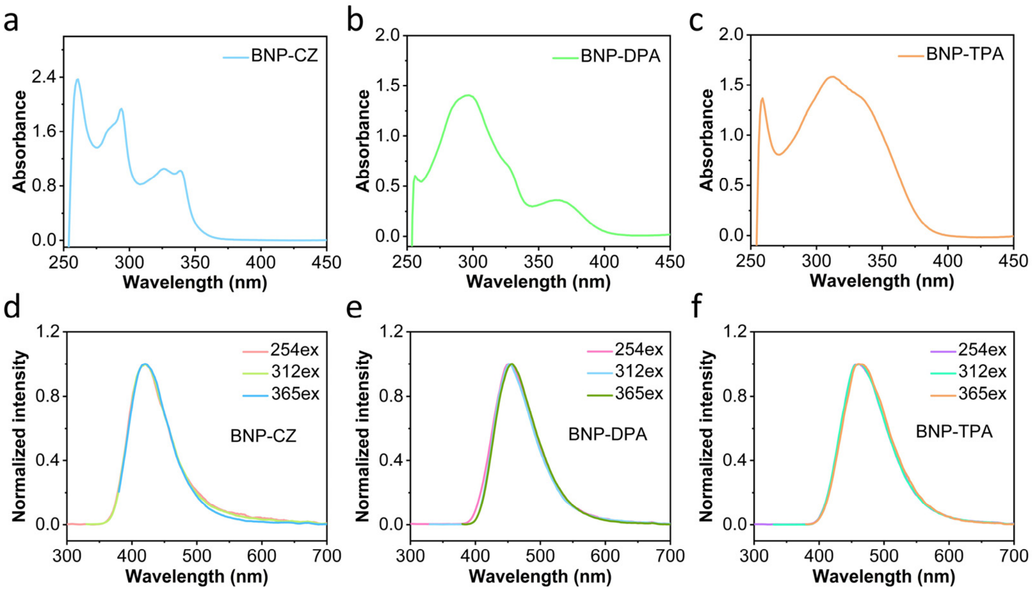

Figure S1. UV-visible absorption spectra of (a) BNP-CZ, (b) BNP-DPA, and (c) BNP-TPA in DMSO solution. (d-f) Prompt PL spectra of BNP-CZ, BNP-DPA, and BNP-TPA chromophores (dissolved in DMSO solution) at different excitation wavelengths.

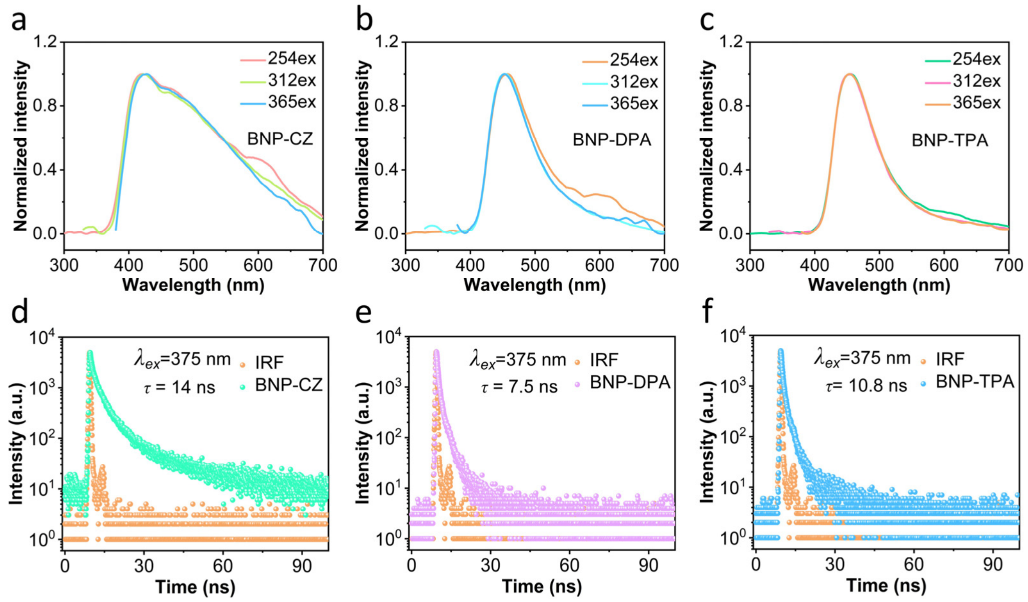

Figure S2. Prompt PL spectra of chromophore powders (a) BNP-CZ, (b) BNP-DPA, and (c) BNP-TPA under different excitations. Time-resolved emission spectra of solid molecular powders (d) BNP-CZ, (e) BNP-DPA, and (f) BNP-TPA, respectively (λ_{ex} = 375 nm).

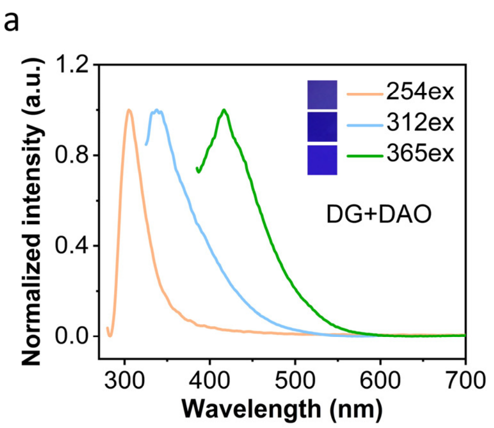

Figure S3. Prompt PL spectra of polymer films cross-linked with (a) DAO, and (b) 1-NA under different excitations. Fluorescence photos corresponding to different UV excitations are also included in the figure.

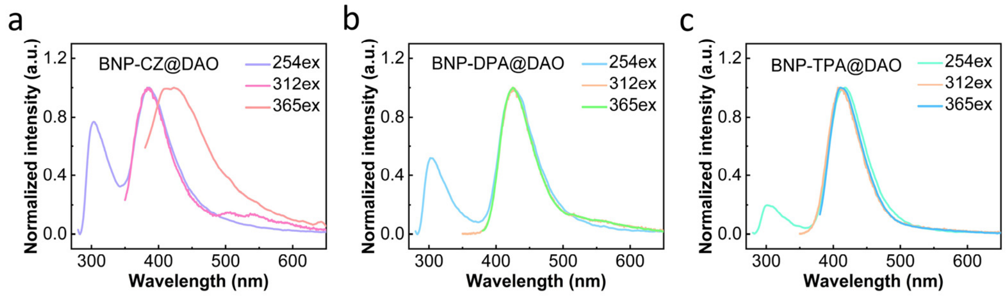

Figure S4. Prompt PL spectra of doped films (a) BNP-CZ@DAO, (b) BNP-DPA@DAO, and (c) BNP-TPA@DAO under different excitations.

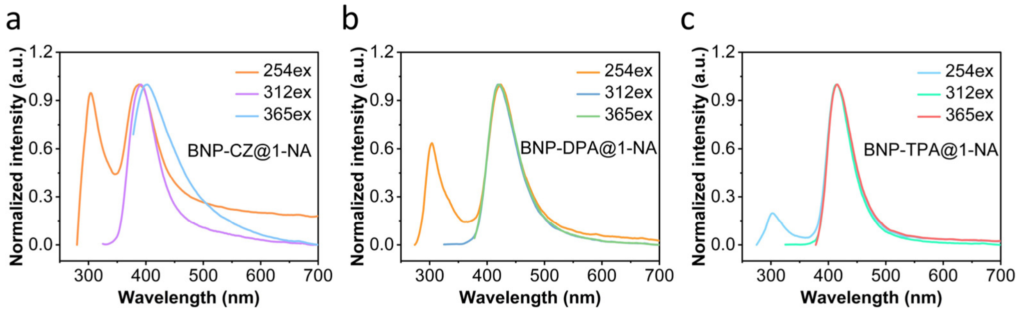

Figure S5. Prompt PL spectra of doped films (a) BNP-CZ@1-NA, (b) BNP-DPA@1-NA, and (c) BNP-TPA@1-NA under different excitations.

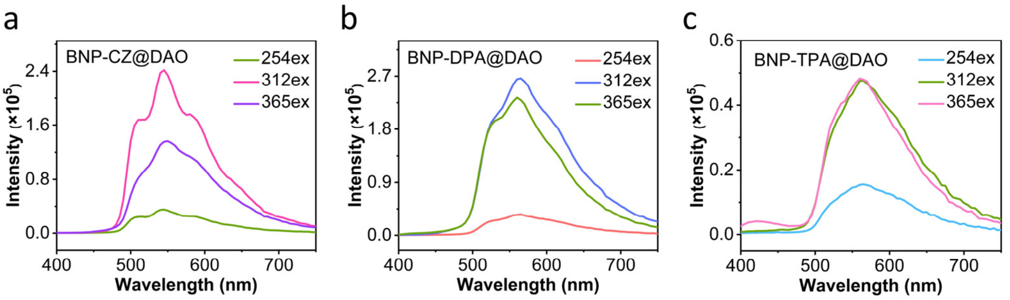

Figure S6. Delayed PL spectra of polymer films (a) BNP-CZ@DAO, (b) BNP-DPA@DAO, and (c) BNP-TPA@DAO under different excitations (t_d=1 ms).

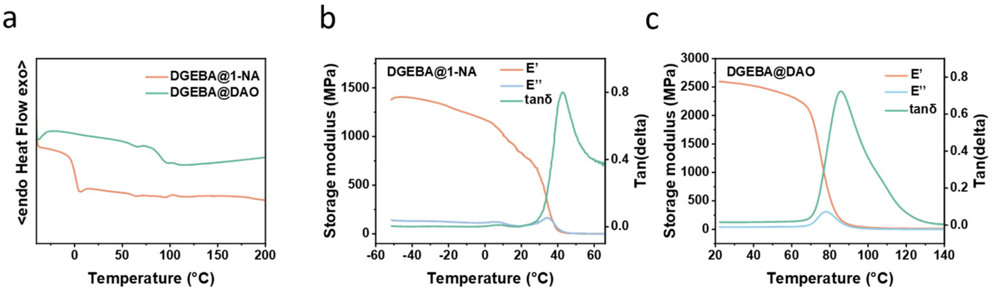

Figure S7. (a) DSC curves of DGEBA cured with different curing agents (1-NA and DAO, respectively). (b-c) DMA curves of DGEBA cured with (b) 1-NA and (c) DAO, respectively.

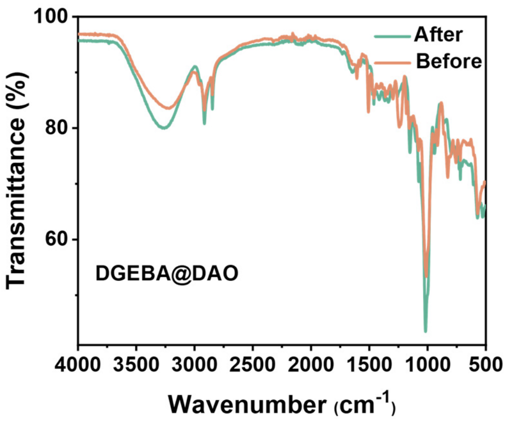

Figure S8. FTIR spectra of DGEBA@DAO before and after thermal curing ("Before" refers to the uncured mixture of DGEBA resin and DAO curing agent, while "After" corresponds to the thermally cured sample).

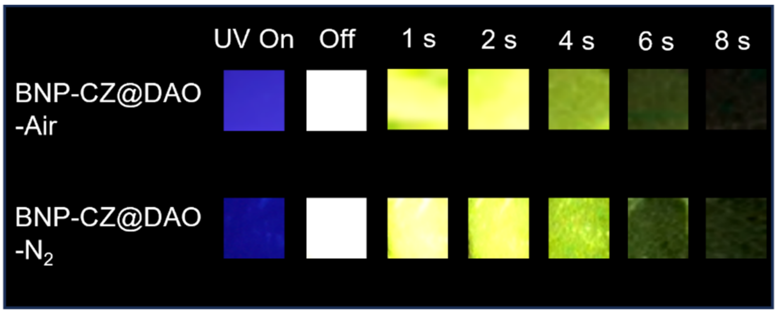

Figure S9. Photographs of BNP-CZ@DAO film in air and nitrogen atmosphere (λ_{ex} = 312 nm).

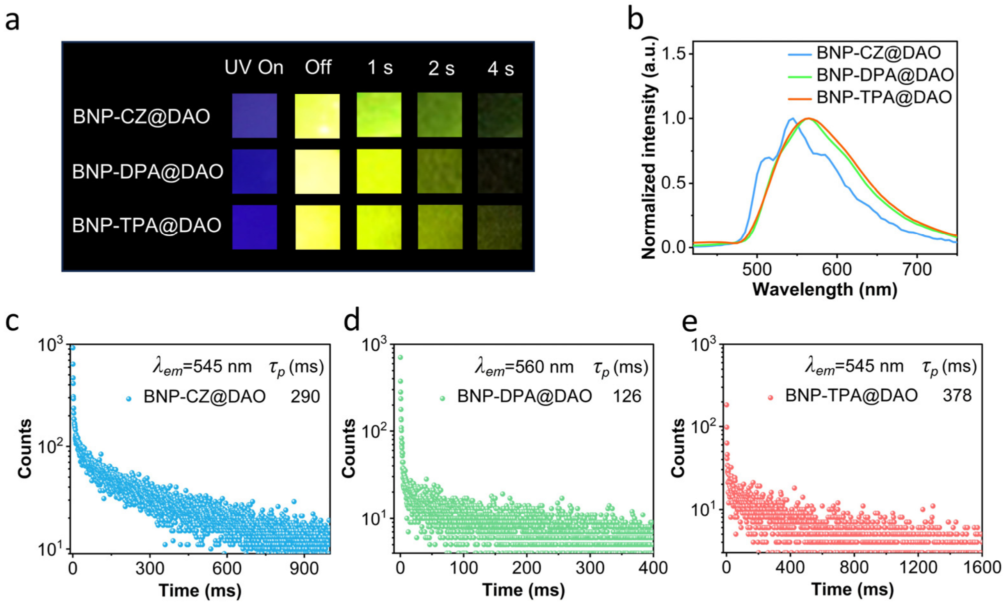

Figure S10. (a) Photographs of polymer films of the DAO system taken under a 254 nm lamp on and off. (b) Normalized delayed PL spectra (t_d = 1 ms) of polymer films under 254 nm excitation. Time-resolved emission spectra of polymer films (c) BNP-CZ@ DAO, (d) BNP-DPA@ DAO, and (e) BNP-TPA@ DAO (λ_{ex} = 254 nm).

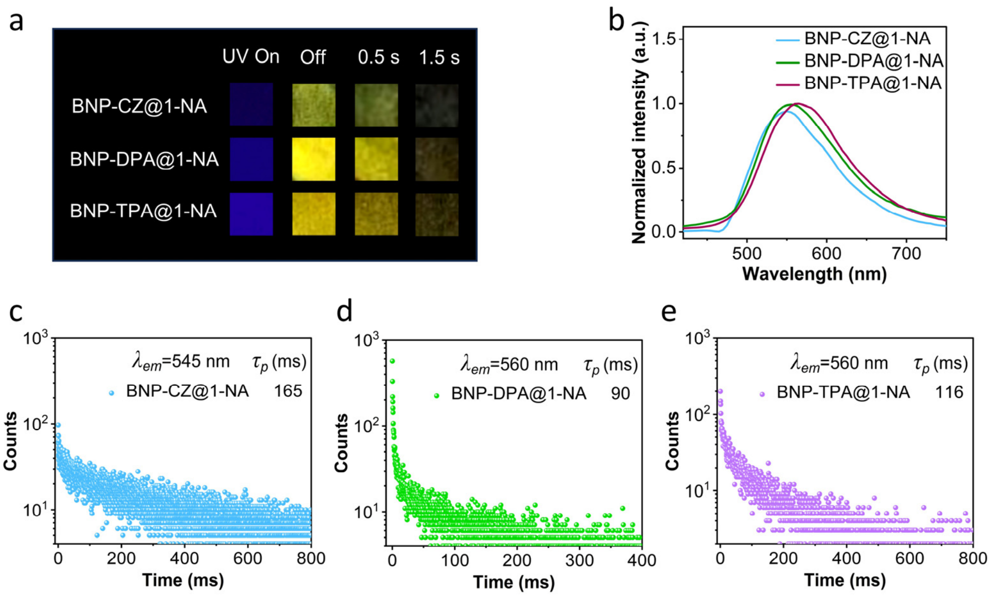

Figure S11. (a) Photographs of polymer films of the 1-NA system taken under a 254 nm lamp on and off. (b) Normalized delayed PL spectra (t_d = 1 ms) of polymer films under 254 nm excitation. Time-resolved emission spectra of polymer films (c) BNP-CZ@1-NA, (d) BNP-DPA@1-NA, and (e) BNP-TPA@1-NA (λ_{ex} = 254 nm).

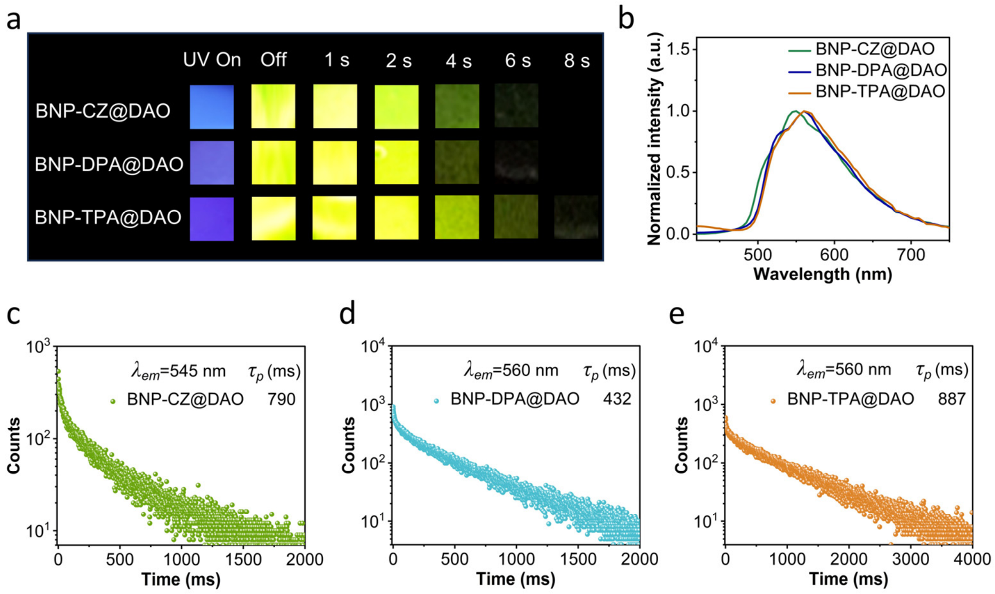

Figure S12. (a) Photographs of polymer films of the DAO system taken under a 365 nm lamp on and off. (b) Normalized delayed PL spectra (t_d = 1 ms) of polymer films under 365 nm excitation. Time-resolved emission spectra of polymer films (c) BNP-CZ@ DAO, (d) BNP-DPA@ DAO, and (e) BNP-TPA@ DAO (λ_{ex} = 365 nm).

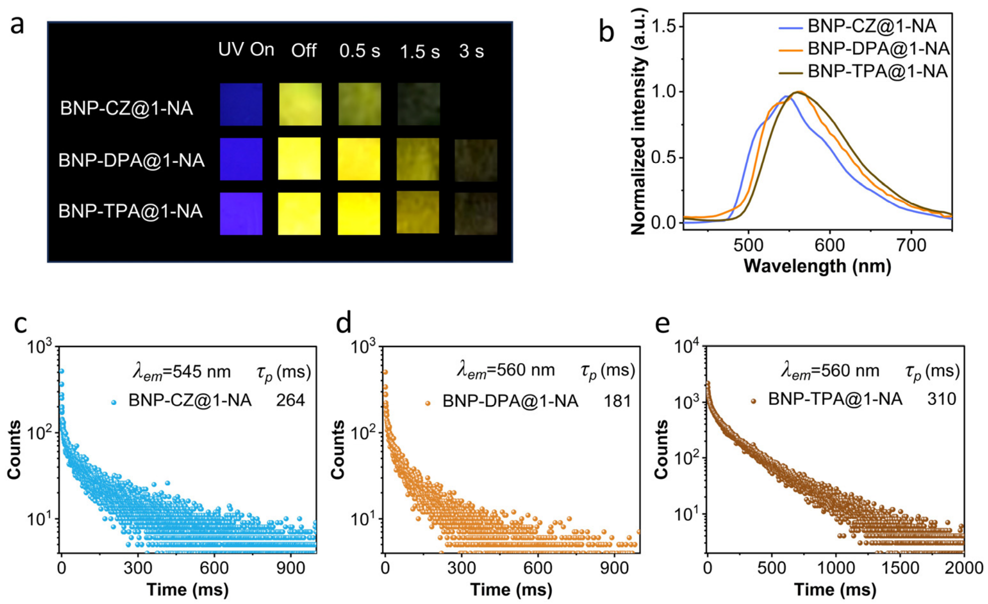

Figure S13. (a) Photographs of polymer films of the 1-NA system taken under a 365 nm lamp on and off. (b) Normalized delayed PL spectra (t_d = 1 ms) of polymer films under 365 nm excitation. Time-resolved emission spectra of polymer films (c) BNP-CZ@1-NA, (d) BNP-DPA@1-NA, and (e) BNP-TPA@1-NA (λ_{ex} = 365 nm).

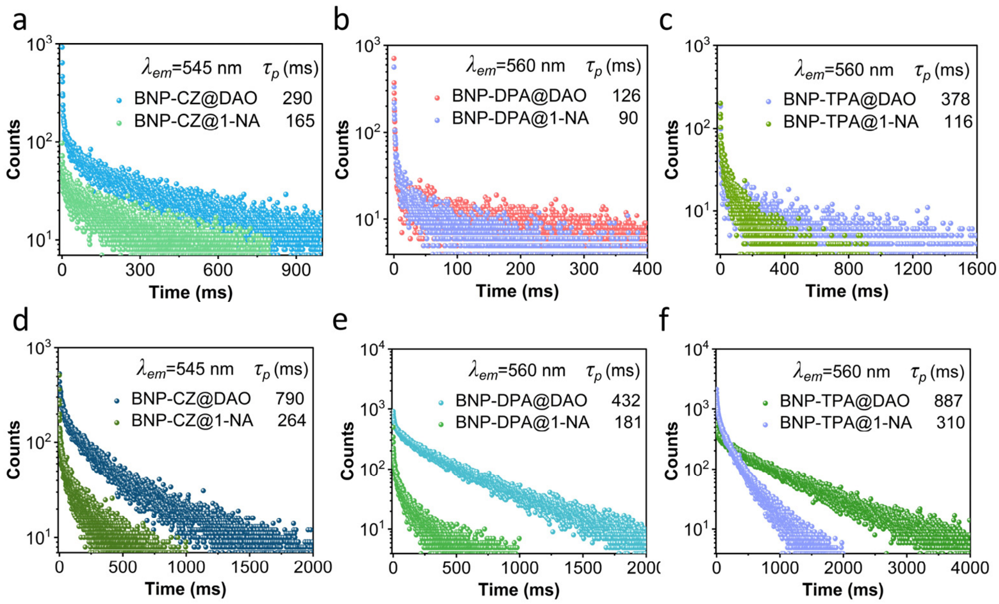

Figure S14. Time-resolved emission spectra of DAO system and 1-NA system polymer films under (a-c) 254 nm and (d-f) 365 nm excitation, respectively.

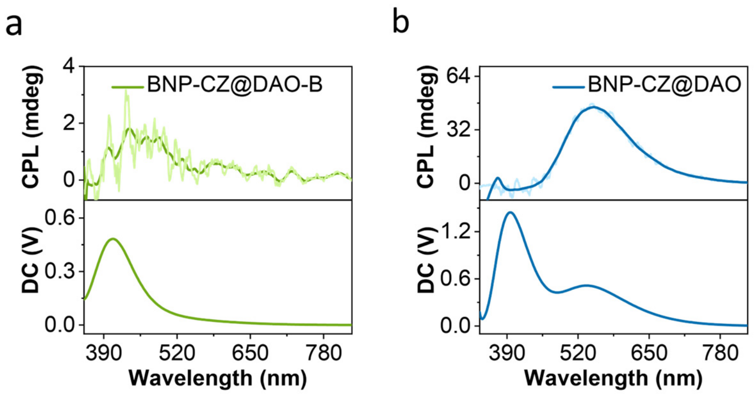

Figure S15. CPL spectra of (a) BNP-CZ@DAO-B (before thermal curing) and (b) BNP-CZ@DAO (after thermal curing).

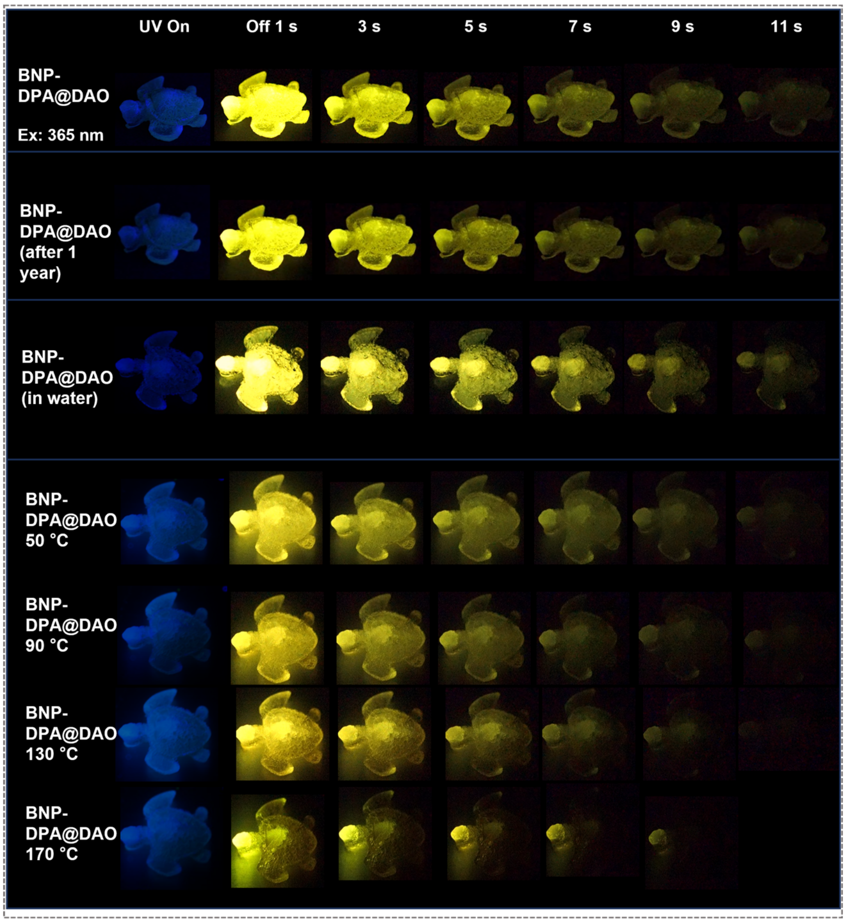

Figure S16. Photographs of BNP-DPA@DAO polymer under different conditions (λ_{ex} = 365 nm).

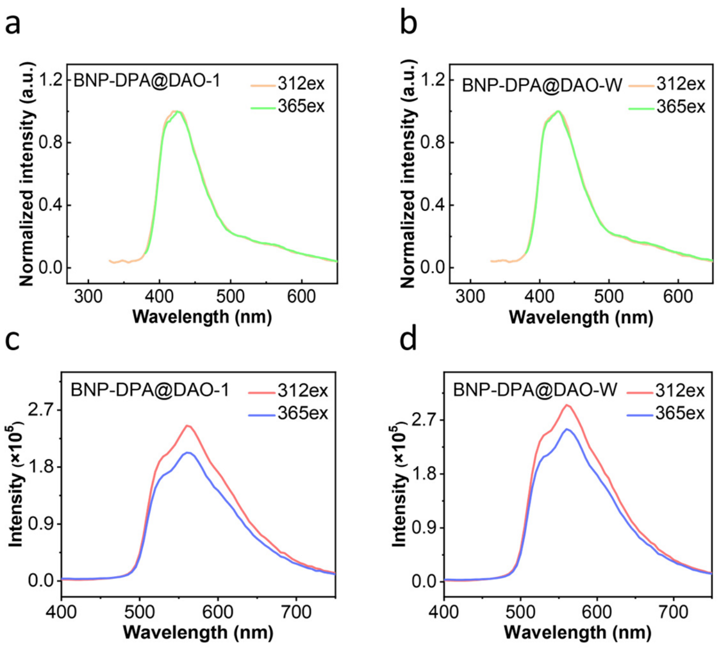

Figure S17. Photoluminescence spectra of BNP-DPA@DAO samples under different conditions: (a, b) normalized prompt PL and (c, d) delayed PL of BNP-DPA@DAO-1 (sample stored for one year) and BNP-DPA@DAO-W (sample after soaked in water), excited at 312, and 365 nm, respectively.

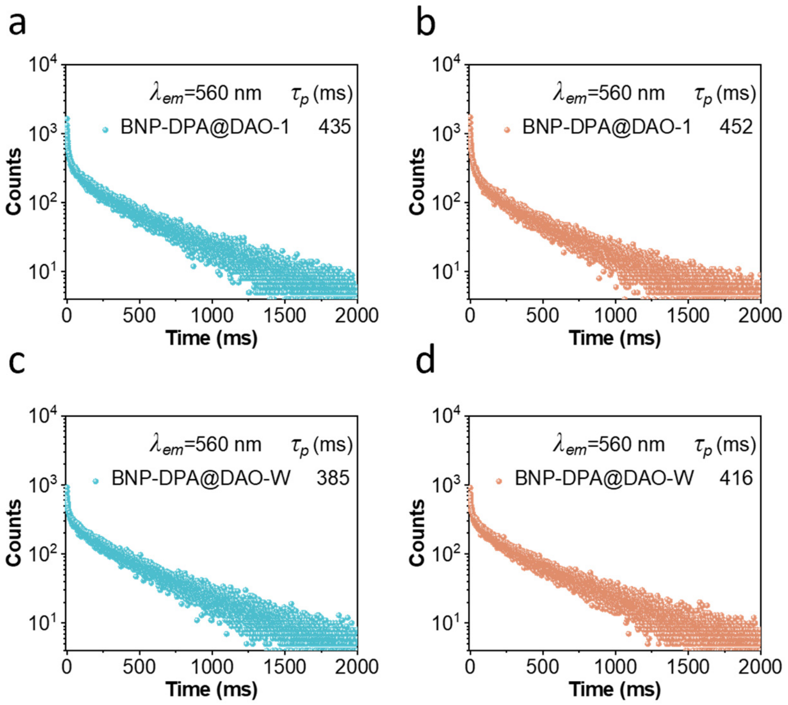

Figure S18. Time-resolved emission spectra of (a-b) BNP-DPA@DAO-1 and (c-d) BNP-DPA@DAO-W under 312 nm, and 365 nm excitation, respectively.

Table S1. The recipe of the doped polymers.

Samples	Composition					
	DGEBA (g)	DAO (g)	1-NA (g)	BNP-CZ (mg)	BNP-DPA (mg)	BNP-TPA (mg)
BNP-CZ@DAO	0.4	0.08	-	0.8	-	-
BNP-DPA@DAO	0.4	0.08	-	-	0.8	-
BNP-TPA@DAO	0.4	0.08	-	-	-	0.8
BNP-CZ@1-NA	0.4	-	0.2	0.8	-	-
BNP-DPA@1-NA	0.4	-	0.2	-	0.8	-
BNP-TPA@1-NA	0.4	-	0.2	-	-	0.8

Table S2. Comparison of photophysical properties of BNP-CZ@DAO with other CPP materials reported in previous literature.

Samples	Photophysical property				
	λ_{em} (nm)	Φ_{PL} (%)	lifetime (ms)	$\lvert g_{lum} \rvert$ ($\times10^{-3}$)	Ref.
CPOA	465	20.54	1680	4.0	[1]
CEC/TBB-6OMe	454	7.0	409	14.0	[2]
P2@xR5011	556	1.84	0.145	160.0	[3]
(R,S,R)/(S,R,S)-Pt2	800	35.4	1250	3.9	[4]
BNP-CZ@DAO	545	5.94	882	5.5	This work

Table S3. Photophysical properties (fluorescence emission maximum wavelength, CPL emission wavelength, and absolute value of the CPL dissymmetry factor $\lvert g_{lum} \rvert$) of the BNP derivatives in DMSO solution.

Samples	Photophysical property		
	λ_{max}(fl) (nm)	CPL (nm)	$\lvert g_{lum, CPF} \rvert$ ($\times10^{-3}$)
BNP-CZ	425	408	0.56
BNP-DPA	454	440	1.47
BNP-TPA	454	450	0.29

Table S4. Photophysical properties of the chiral doped polymer films, including the peak wavelengths of circularly polarized fluorescence (CPF) and circularly polarized phosphorescence (CPP), phosphorescence lifetime τ_P, photoluminescence quantum yield Φ, and the corresponding luminescence dissymmetry factors ($|g_{lum}|$) under excitation at 312 nm.

Samples	Photophysical property					
	CPF (nm)	CPP (nm)	τ_P (ms)	Φ (%)	$\lvert g_{lum, CPF} \rvert$ ($\times 10^{-3}$)	$\lvert g_{lum, CPP} \rvert$ ($\times 10^{-3}$)
BNP-CZ@DAO	395	535	882	5.94	0.38	5.56
BNP-DPA@DAO	430	560	437	20.22	1.71	1.61
BNP-TPA@DAO	420	560	973	26.55	0.16	0.47
BNP-CZ@DAO-B	410	550	-	-	0.41	0.03

Table S5. Phosphorescence lifetimes of different samples.

λ_{ex} [nm]	λ_{em} [nm]	A_1 [%]	$<\tau>_1$ [ms]	A_2 [%]	$<\tau>_2$ [ms]	A_3 [%]	$<\tau>_3$ [ms]	χ^2
BNP-CZ@DAO								
254	545	8.66	24.3	91.34	290.0	-	-	1.138
312	545	2.15	32.0	16.20	198.1	81.65	882.0	1.090
365	545	5.25	23.3	32.33	189.3	62.42	790.0	1.145
BNP-DPA@DAO								
254	560	7.69	3.4	92.31	126.0	-	-	1.254
312	560	3.26	5.5	12.71	67.3	84.03	437.0	1.171
365	560	9.48	51.3	90.52	432.0	-	-	1.178
BNP-TPA@DAO								
254	560	0.30	1.3	4.84	21.9	94.85	378.0	1.208
312	560	2.05	34.3	14.43	305.2	83.52	973.0	1.136
365	560	5.78	90.9	94.92	887.0	-	-	1.157
BNP-CZ@1-NA								
254	545	100	165.0	-	-	-	-	1.281
312	545	13.04	38.1	86.96	287.0	-	-	1.236
365	545	3.68	6.2	19.19	49.4	77.13	264.0	1.215
BNP-DPA @1-NA								
254	560	16.08	6.1	83.92	90.0	-	-	1.125
312	560	8.91	8.7	21.90	47.8	69.19	160.0	1.127
365	560	4.86	6.9	24.45	35.2	70.69	181.0	1.101
BNP-TPA@1-NA								
254	560	17.16	14.7	82.84	116.0	-	-	1.003
312	560	24.28	33.4	75.72	204.0	-	-	1.013
365	560	5.80	14.0	28.33	89.8	65.88	310.0	1.159
BNP-DPA@DAO-1								
312	560	12.07	72.2	87.93	435.0	-	-	1.212
365	560	5.82	13.4	18.43	100.1	75.74	452.6	1.164

BNP-DPA@DAO-W								
312	560	8.45	46.3	91.55	385.0	-	-	1.137
365	560	10.16	35.8	89.84	416.0	-	-	1.289

Notes: data for this table were calculated according to the methodology reported in prior literature [5].

References

1. Sun, Y.; Zheng, L.; Shu, Y.; Song, Y.; Chen, H.; Zhao, L.; Chang, J.; Xin, P. J. A. O. M. Full-Color Circularly Polarized Organic Afterglow Enabled by Tunable Triplet-State Chirality via a Cascade Confinement Strategy. *Adv. Opt. Mater.* **2026**. e71286

2. You, J.; Tian, R.; Yin, C.; Wang, J.; Zhang, J.; Zhang, J. Organic Circularly Polarized Room-Temperature Phosphorescence Toolbox with Excellent Practicality and Functionality. *ACS Nano* **2025**, *19* (44), 38219-38230.

3. Li, J.; Guan, Y.; Hao, T. T.; Huang, J.; Chen, Y.; Li, H.; Duan, P.; Xie, H.-L. Phosphorescent Liquid Crystalline Polymer-based Circularly Polarized Luminescence Optical Waveguides for Enhanced Photonic Signal Processing and Information Encryption. *Angew. Chem. Int. Ed.* **2025**, *64*.

4. Gu, S.; Li, D.; Long, D.; Yu, X.; Li, W.; Ma, S.; Tao, P. Unlocking efficient near-infrared circularly polarized phosphorescence reaching 800 nm in cyclometalated Pt(ii) complexes. *Chem. Commun.* **2026**, *62* (39), 9904-9908.

5. Wang, Z.; Li, A.; Zhao, Z.; Zhu, T.; Zhang, Q.; Zhang, Y.; Tan, Y.; Yuan, W. Z. Accessing Excitation- and Time-Responsive Afterglows from Aqueous Processable Amorphous Polymer Films through Doping and Energy Transfer. *Adv. Mater.* **2022**, *34* (31), 2202182.
